# Correction: Host and environmental determinants of microbial community structure in the marine phyllosphere

**DOI:** 10.1371/journal.pone.0259357

**Published:** 2021-10-26

**Authors:** Margaret A. Vogel, Olivia U. Mason, Thomas E. Miller

The following information is missing from the Funding statement: This research was supported in part by DEB-1456425 from the US National Science Foundation.
